# Supplementary material for: Loss of tomato geranylgeranyl diphosphate synthase 2 increases monoterpenoid levels and enhances immune responses to bacterial infection
Source: bioRxiv. 2025 Mar 26:2025.03.24.644926. Preprint. [Version 1] doi: 10.1101/2025.03.24.644926 (PMC11974751; doi:10.1101/2025.03.24.644926)
Supplement: Supplement 1 [file NIHPP2025.03.24.644926v1-supplement-1.pdf]

bioRxiv preprint doi: https://doi.org/10.1101/2025.03.24.644926; this version posted March 26, 2025. The copyright holder for this preprint (which was not certified by peer review) is the author/funder, who has granted bioRxiv a license to display the preprint in perpetuity. It is made available under aCC-BY 4.0 International license.

SUPPLEMENTAL INFORMATION

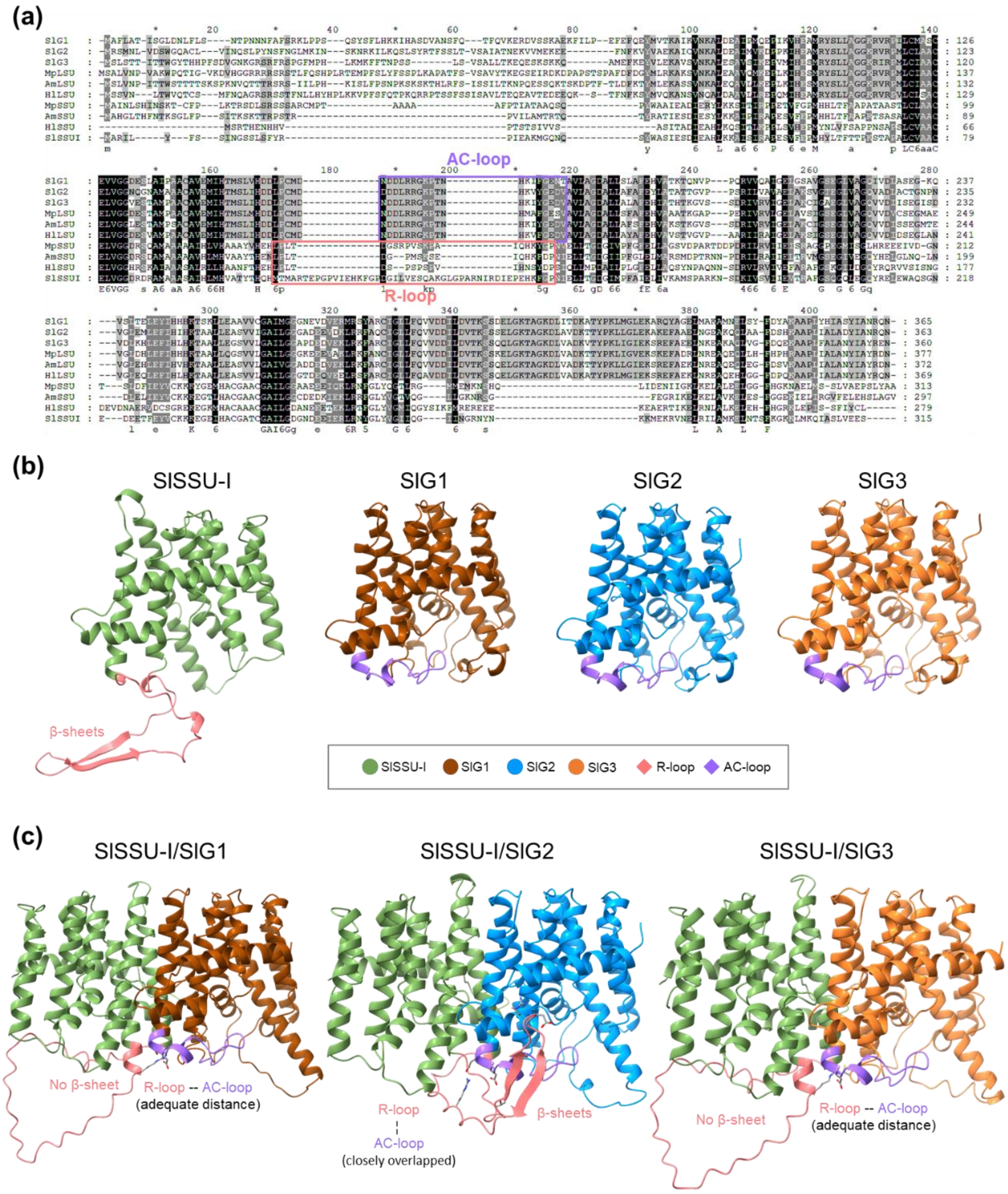

Supplementary Fig. S1. Sequence alignment and protein structure prediction. (a) Amino acid sequence alignment: SlG1 (*Solanum lycopersicum* GGPPS1), SlG2 (*S. lycopersicum* GGPPS2), SlG3 (*S. lycopersicum* GGPPS3), MpLSU (*Mentha piperita* LSU), AmLSU (*Antrirrhinum majus* LSU), HlLSU (*Humulus lupulus* LSU), MpSSU (*M. piperita* SSU), AmSSU (*A. majus* SSU), HlSSU (*H. lupulus* SSU), SlSSUI (*S. lycopersicum* SSU-I). All sequences presented here have the N-terminal signal peptides omitted. (b) Predicted monomeric protein structures using AlphaFold3. (c) Predicted heterodimeric protein structures using AlphaFold3. All protein structures were visualized with ChimeraX.

bioRxiv preprint doi: https://doi.org/10.1101/2025.03.24.644926; this version posted March 26, 2025. The copyright holder for this preprint (which was not certified by peer review) is the author/funder, who has granted bioRxiv a license to display the preprint in perpetuity. It is made available under aCC-BY 4.0 International license.

Supplementary Table S1. PDBePISA analysis of interface summary between tomato SSU-I and SlG1, SlG2, or SlG3 in heterodimers

Interfacing value		SlSSU-I/SlG1 heterodimer				**SlSSU-I/SlG2 heterodimer**				SlSSU-I/SlG3 heterodimer			
		SlSSU-I		SlG1		**SlSSU-I**		**SlG2**		SlSSU-I		SlG3	
Number of atoms	Interface	179	7.9%	178	8.1%	**202**	**9.0%**	**214**	**9.8%**	179	7.9%	181	8.1%
	Surface	1372	60.8%	1253	56.8%	1351	59.9%	1252	57.3%	1378	61.1%	1292	57.6%
	Total	2255	100.0%	2207	100.0%	2255	100.0%	2186	100.0%	2255	100.0%	2244	100.0%
Number of residues	Interface	50	17.2%	50	17.2%	**56**	**19.2%**	**62**	**21.1%**	49	16.8%	47	15.9%
	Surface	259	89.0%	263	90.4%	261	89.7%	266	90.5%	260	89.3%	256	86.5%
	Total	291	100.0%	291	100.0%	291	100.0%	294	100.0%	291	100.0%	296	100.0%
Solvent-accessible area (Å²)	Interface	1939.7	11.2%	1922.9	13.2%	**2165.6**	**13.1%**	**2195.5**	**15.3%**	1972.2	11.5%	1945.6	12.7%
	Total	17359.5	100.0%	14557.1	100.0%	16481.5	100.0%	14396.2	100.0%	17169.8	100.0%	15323.8	100.0%
Solvation energy (kcal/mol)	Isolated structure	-244.6	100.0%	-278.6	100.0%	-249	100.0%	-275.8	100.0%	-246.2	100.0%	-281.4	100.0%
	Gain on complex formation	-16	6.6%	-19.4	7.0%	**-21.4**	**8.6%**	**-21.6**	**7.8%**	-16.4	6.7%	-20.4	7.3%
	Average gain	-6.6	2.7%	-4.8	1.7%	**-6.7**	**2.7%**	**-8.2**	**3.0%**	-6.4	2.6%	-5.8	2.1%
	P-value	0.021		0.000		0.001		0.002		0.015		0.001	

Suplementary Table S2. Listed qPCR primers used.

**Primer Name**	**Forward Sequence**	**Reverse Sequence**
**SLG1**	GGCCTTTGAACATGTGGCTACC	ACTCGCCAAGTCCACAATTTGC
**SLG3**	AGGAGGTGCACCAGATGAAG	TCAGCAACCAAGTCCTTCCC
**SSU-I**	GGACAGCTAGAAGGCCAATATC	GCTCCACATGCATGAATTTCC
**SSU-II**	CGAGTCCTCCGGGTTATTA	CACTCACCCATTTCACCATATTTC
**GGPPS**	AGTTCAGAGATCAAGACGAGC	GCTGCACCTGATAGATTCCC
**ICS**	TGCCTCATGGACATACCAGA	TATGCGAATGGGGATTTTTTC
**Actin**	CTAGGCTGGGTTCGCAGGAGATGATGC	GTCTTTTTGACCCATACCCACCATCACAC
